# Oligomerization of bacterially expressed H1N1 recombinant hemagglutinin contributes to protection against viral challenge

**DOI:** 10.1038/s41598-018-30079-4

**Published:** 2018-08-07

**Authors:** Tess E. Kuenstling, Anthony R. Sambol, Steven H. Hinrichs, Marilynn A. Larson

**Affiliations:** 1 Department of Pathology & Microbiology, University of Nebraska Medical Center, Omaha, NE USA; 2 Nebraska Public Health Laboratory, Omaha, NE USA

## Abstract

Vaccination is the most effective intervention to prevent influenza and control the spread of the virus. Alternatives are needed to the traditional egg-based vaccine strategy for a more rapid response to new outbreaks. Two different hemagglutinin (HA) fragments (rHA1_1-326_ and rHA1_53-269_) derived from influenza A virus subtype H1N1 were expressed in *Escherichia coli* and characterized by immunoblot, gel filtration, hemagglutination, and competitive binding assays. rHA1_1-326_ included neutralizing epitopes and the trimerization domain, whereas rHA1_53-269_ included only the head of HA with the neutralizing epitopes. Mice were immunized with rHA1_1-326_ or rHA1_53-269_, and sera were tested for the presence of neutralizing antibodies. Mice were then challenged with H1N1 and infection severity was monitored. rHA1_1-326_ trimerized, whereas rHA1_53-269_ was unable to form oligomers. Both rHA1_1-326_ and rHA1_53-269_ elicited the production of neutralizing antibodies, but only oligomerized rHA1_1-326_ protected against live virus challenges in mice. This study demonstrated that bacterially expressed HA was capable of folding properly and eliciting the production of neutralizing antibodies, and that HA oligomerization contributed to protection against viral challenge. Therefore, prokaryotic-derived vaccine platforms can provide antigenic and structural requirements for viral protection, as well as allow for the rapid and cost-effective incorporation of multiple antigens for broader protection.

## Introduction

Influenza seasonal infections lead to approximately 36,000 deaths in the United States alone each year with an associated annual economic burden of $87.1 billion dollars^1,2^. Vaccinations are the primary method employed to control the seasonal spread of the influenza virus, as well as aid in pandemic preparedness. Almost all current influenza vaccines utilize a lengthy egg-based vaccine manufacturing process, requiring a minimum of five months to generate the vaccine^3^. Additional drawbacks to current egg-based vaccine manufacturing include the vulnerability of chicken populations to disease and limited scale-up capacity. Recent studies have also shown that the virus mutates to adapt to growth in the egg^4^, contributing to antigenicity that differs from circulating viral strains by the time the vaccine is ready for use. Therefore, a more rapid process that increases the efficiency and availability of an influenza vaccine is needed.

Seasonal vaccine strains for the northern hemisphere are selected at least six months before the flu season starts. This can result in a mismatch of the vaccine and circulating strains from antigenic drift, resulting in poor protection from the influenza virus^5^. For example, during the 2007–2008 influenza season, A/Wisconsin (H3N2) was selected as the strain to be included in the vaccine, yet A/Brisbane (H3N2) virus became the dominant circulating strain; therefore, the available vaccine provided no protection^6^. Lengthy manufacturing times also impact responsiveness to emerging strains and antigenic shift in the pandemic strains. The 2009 H1N1 pandemic vaccine was released six weeks behind schedule due to manufacturing delays^7^. If vaccines could have been distributed just one month earlier, an estimated 2,200 lives would have been saved in the United States^8^.

**Figure 1:**
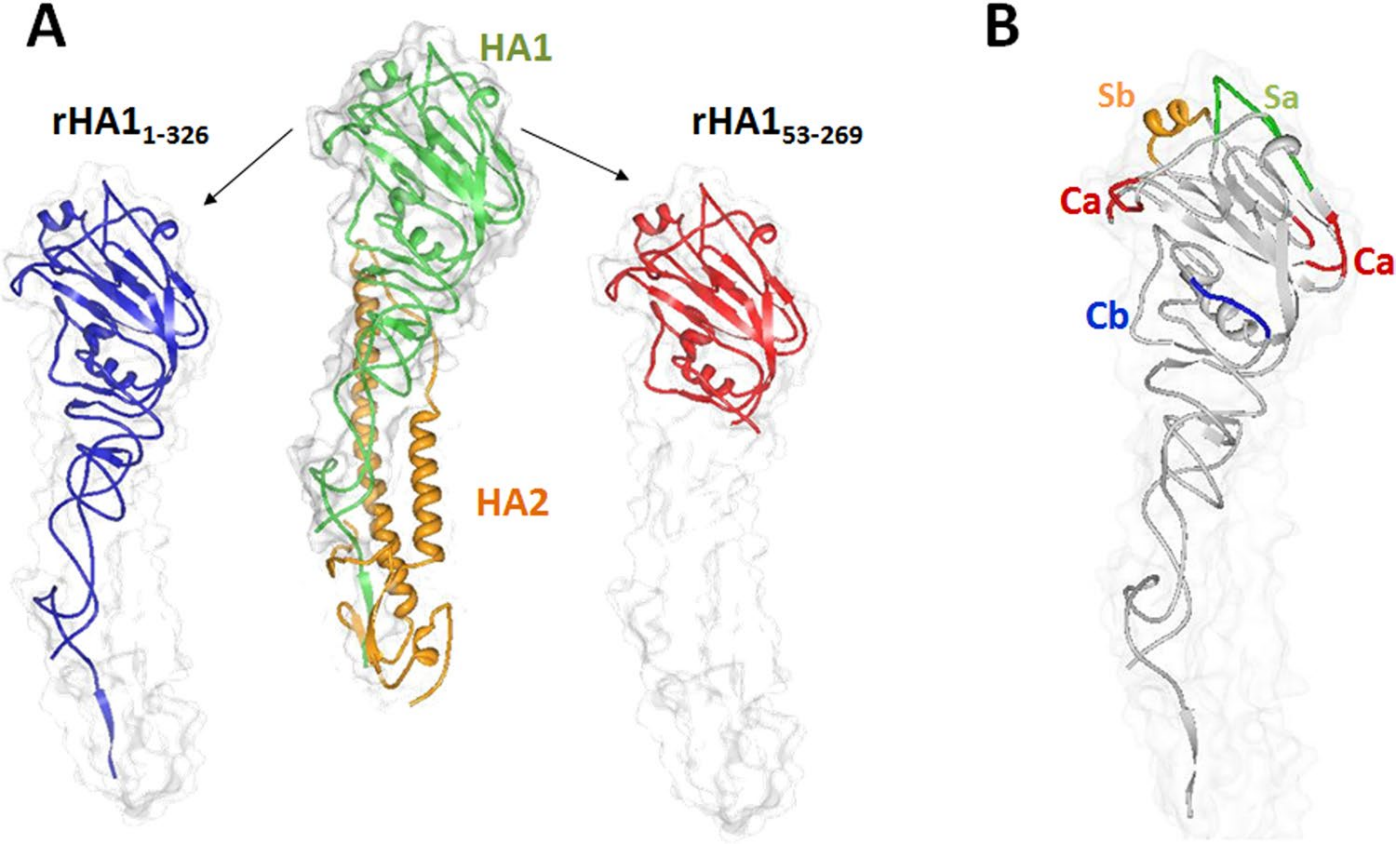
Structural representations of rHA1_1-326_ and rHA1_53-269_. (**A**) Crystal structure models of rHA1_1-326_ and rHA1_53-269_ containing residues 1-326 and 53-269, respectively, are shown and were obtained from the Research Collaboratory for Structural Bioinformatics (RCSB) Protein Data Bank (PDB)^21^, using the Visualize 3D Viewer (www.rcsb.org) for PDB ID 3AL4. The PDB ID 3AL4 crystal structure was derived from swine-origin A (H1N1)-2009 influenza A virus hemagglutinin^22^. (**B**) The relative location of the H1N1 neutralizing epitopes (Ca, Cb, Sa, and Sb) are shown, and both rHA1_1-326_ and rHA1_53-269_ contained these regions.

For influenza, hemagglutinin (HA) is the primary viral protein recognized by the immune system and subsequently is the primary target for vaccine design^9^. HA is composed of two subunits, HA1 and HA2 (Fig. 1). HA1 consists of a globular head, which is responsible for receptor binding and contains neutralizing epitopes Ca1, Ca2, Cb, Sa, and Sb. HA2 is composed of a stem structure that supports HA1 and mediates membrane fusion during viral entry. Trimerization of HA is required for complete antigenicity of epitopes Ca1, Ca2, Cb, and Sa^10^.

Alternatives to the egg-based production of influenza vaccines include the use of mammalian cell culture or baculovirus expression systems, since appropriate glycosylation of specific epitopes is often needed in order to generate protective antibodies against the infecting pathogen. Therefore, improvements in vaccine manufacturing have typically focused on these expression platforms, due to the belief that post-translation modifications (e.g., glycosylation) were critical for producing HA antigens that elicited neutralizing antibodies^11^. Although investigations to determine the functional role of HA glycosylation are ongoing, others have shown that glycosylation of HA is not required for generating protective antibodies^12^, prompting our investigation of a recombinant HA vaccine using a prokaryotic expression system.

Advantages of vaccine generation utilizing a prokaryotic expression platform include rapid production times, readily amendable genetics, and economic savings over cell culture expression systems. Several studies have demonstrated that bacterial expression and subsequent immunization with recombinant HA from H5N1 conferred protection in animal models^13–15^. These findings suggested that bacterially expressed HA immunogens may provide an alternative to egg-based vaccines, especially when folded properly and combined with the appropriate adjuvant. The fast production of large amounts of HA-based vaccines that were derived from a bacterial expression platform may provide timely protection against seasonal influenza and newly emerging pandemic influenza outbreaks. However, essential elements or critical domains of H1N1 have yet to be defined for viral protection, especially when using a prokaryotic expression platform. The herein study reports several critical factors that are required to confer protection when using bacterially expressed HA1 from H1N1.

## Results

### Cloning and expression of the recombinant HA proteins

Trimerization of HA is hypothesized to be essential for the complete antigenicity of epitopes Ca1, Ca2, Cb, and Sa and the effective generation of protective antibodies. Residues 63–286 have been shown to elicit the production of neutralizing antibodies due to a functional binding site; however, they do not form functional trimers *in vitro*^16^. Structural analyses identified the location of conserved cysteines outside of this region (amino acids 4, 42, 275, 279, and 303), which were incorporated into a recombinant HA protein that was evaluated in this study.

To investigate whether oligomerization of HA monomers would contribute to the overall effectiveness of a recombinant vaccine, two constructs were generated from the influenza A virus subtype H1N1 strain A/California/04/2009 (Fig. 1A). The first recombinant HA construct, rHA1_1-326_, contained residues 1–326, and the second recombinant HA construct, rHA1_53-269_, was comprised of only residues 53–269. Both recombinant proteins were expressed in the soluble fraction with a typical recovery of 2–3 g/L of purified protein. To determine the contribution of oligomerization for enhancing protection, both constructs contained all previously characterized Sa, Sb, Ca1, Ca2, and Cb H1N1 neutralizing epitopes (Fig. 1B).

### Characterization of rHA1 proteins by immunoblot and gel filtration chromatography

**Figure 2:**
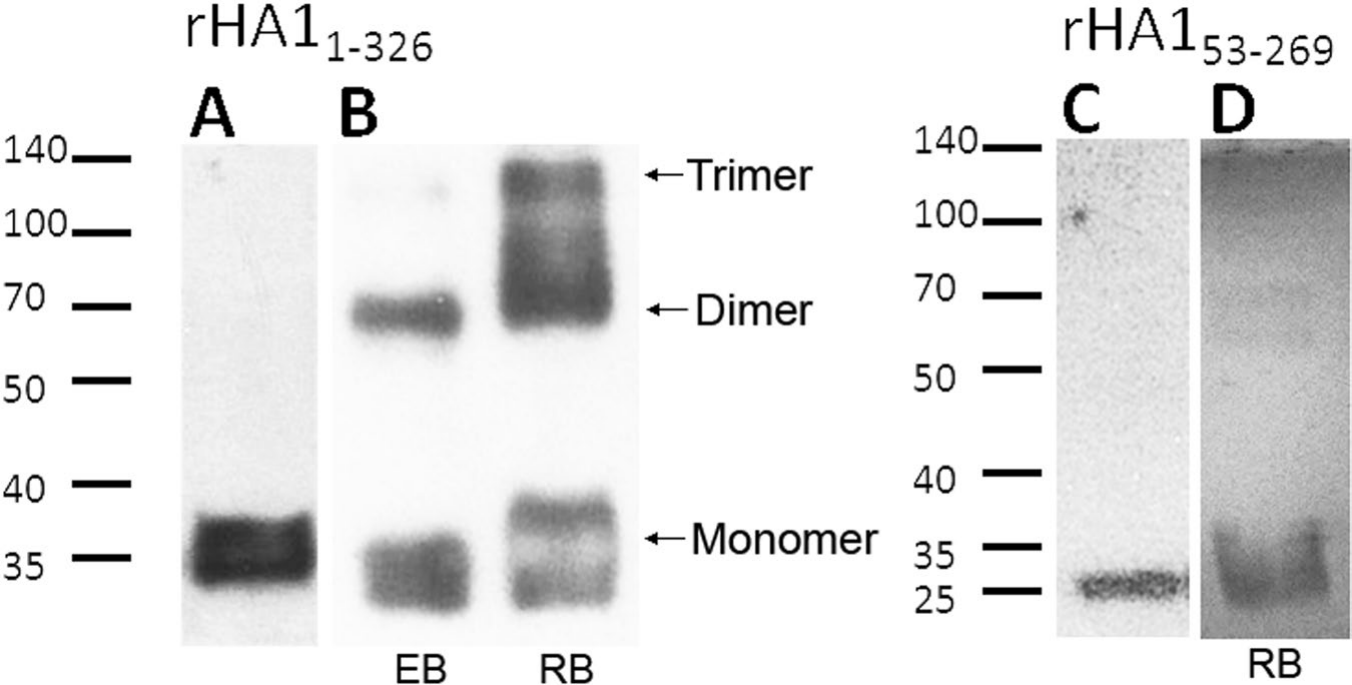
Detection of H1N1 recombinant HA1 oligomers. Immunoblot analyses of the rHA1_1-326_ and rHA1_53-269_ purified proteins were performed under denaturing and non-denaturing conditions with subsequent detection using an anti-His antibody. (**A**) Electrophoretic mobility of rHA1_1-326_ (100 ng) with a predicted MW of 38.6 kDa under denaturing conditions is shown. (**B**) Analysis of rHA1_1-326_ (100 ng) under non-denaturing conditions in elution buffer (EB), and also after buffer exchange in refolding buffer (RB) are shown. (**C**) Analysis of rHA1_53-269_ (40 ng) with a predicted MW of 27.3 kDa under denaturing conditions, and (**D**) under non-denaturing conditions, after buffer exchange in refolding buffer are shown. Molecular weight size markers in kDa are indicated to the left of panels A and C for the denaturing gels. The recombinant HA1 proteins were ran in separate gels under denaturing and non-denaturing conditions, and the uncropped immunoblots of these purified proteins are shown in Supplementary Fig. S1. EB = Elution Buffer, RB = Refolding Buffer.

Both rHA1_53-269_ and rHA1_1-326_ were purified by affinity chromatography and evaluated for their ability to form higher order oligomers, including dimers and trimers. For immunoblot analysis, these recombinant HA1 proteins were probed with an anti-His antibody under denaturing and non-denaturing conditions (Fig. 2A–D). A panel of commercially available buffers designed to assist in protein folding was used to evaluate protein oligomerization under non-denaturing conditions. The refolding buffer that was identified to generate optimal oligomerization of rHA1_1-326_ contained 1.1 M guanidine, 440 mM L-arginine, 55 mM Tris, 21 mM NaCl, 0.88 mM KCl, 1 mM EDTA, 1 mM glutathione, and 1 mM glutathione disulfide at a pH of 8.2. Following buffer exchange of rHA1_1-326_ with the refolding buffer, there was a significant shift to dimeric and trimeric forms of the protein (Fig. 2A,B). However, no oligomerization of rHA1_53-269_ occurred in this refolding buffer nor any of the other buffers tested, and was present only as a monomeric species under both denaturing (Fig. 2C) and non-denaturing conditions following refolding buffer exchange (Fig. 2D).

**Figure 3:**
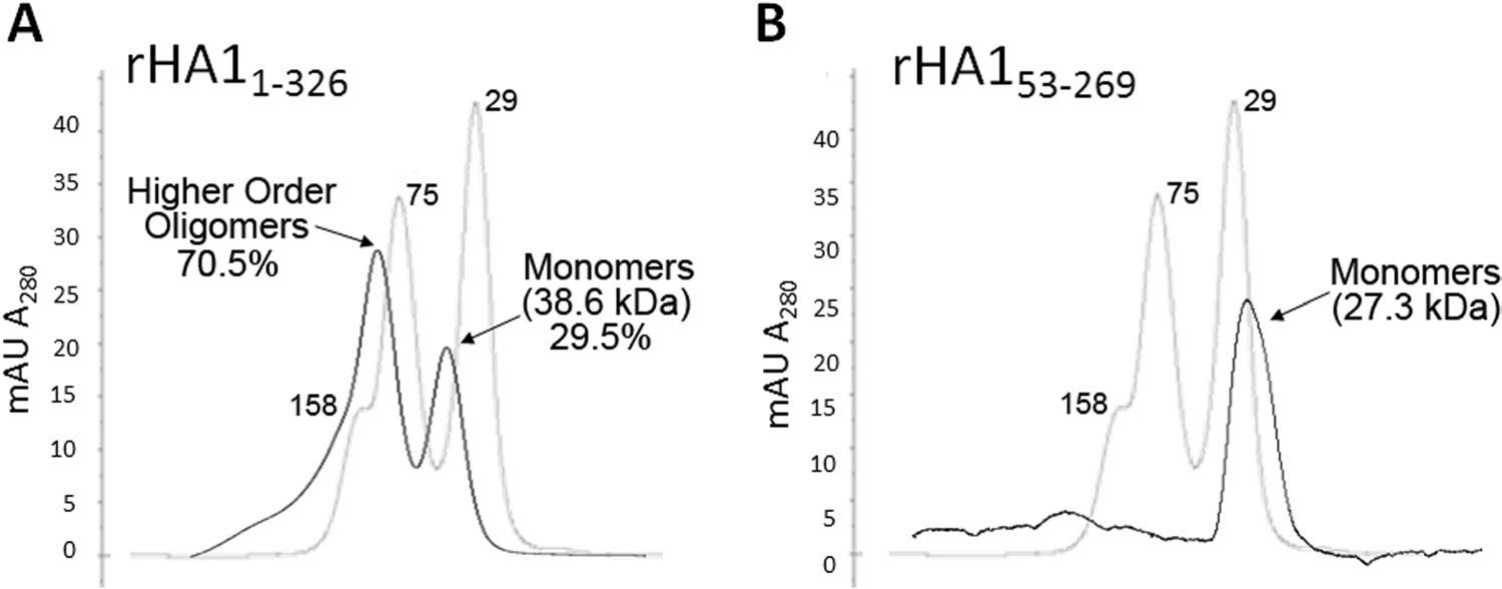
Characterization of purified H1N1 rHA proteins by gel filtration chromatography. Chromatograms of (**A**) rHA1_1-326_ and (**B**) rHA1_53-269_ purified proteins in PBS, after treatment with refolding buffer and subsequent gel filtration on a Sephacryl 16/60 S-300 HR column are shown. Calibration standards (GE, Gel Filtration Calibration Kits LMW 28-4038-41 and HMW 28-4038-42) with 158, 75, and 29 kDa molecular weight markers were used and are denoted with the associated peak in the chromatograms depicted in gray.

Gel filtration chromatography was used as a second method to characterize the composition of higher order oligomers in rHA1_1-326_. Using this approach rHA1_1-326_ dimers and trimers were found to comprise 70.5% of the total recombinant protein species, whereas monomers comprised 29.5% (Fig. 3A), as determined by quantification of the area under the relevant peak. When rHA1_53-269_ was similarly analyzed, there was one dominant peak consistent with an HA monomer of 27.3 kDa (Fig. 3B). These gel filtration results supported the findings obtained for the immunoblot analyses, demonstrating that the rHA1_1-326_ proteins were primarily higher order oligomers, whereas rHA1_53-269_ was monomeric.

### Functional binding by rHA11–326 and rHA153-269 to sialic acid on red blood cells

**Figure 4:**
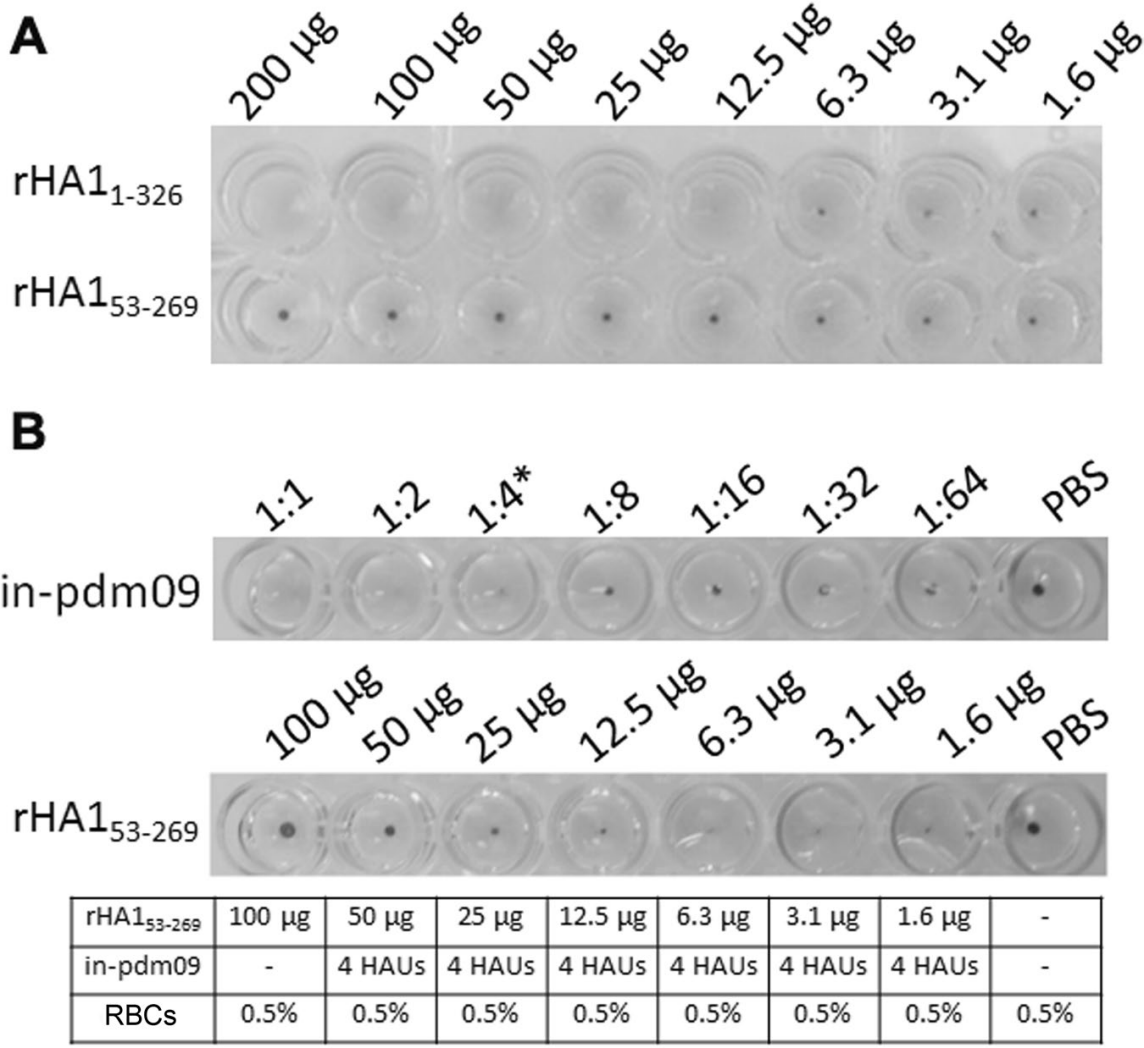
Characterization of rHA1_1-326_ and rHA1_53-269_ functional binding *in vitro*. (**A**) Hemagglutination assay using 0.5% RBCs in the presence of titered recombinant HA1 (200 μg to 1.6 μg per well with rHA1_1-326_ or rHA1_53-269_) is shown. The lack of agglutination results in the pellet formation of the RBCs. (**B**) Competitive binding assay with rHA1_53-269_ (100 μg to 1.6 μg/well) that was serially diluted 1:2, incubated with 0.5% RBCs, and then incubated with 4 HAUs of standardized and inactivated H1N1 (in-pdm09) is shown. Top panel shows the serial dilutions of in-pdm09 and incubation with 0.5% RBCs that establishes the highest dilution whereby HA could be visualized, which was 1:4 and is denoted by an asterisk (*); since the inverse of the dilution is the titer, in-pdm09 contains 4 hemagglutination units (HAUs). Bottom panel shows the standardized volume of 4 HAUs of in-pdm09 mixed with titered rHA1_53-269_ that ranged from 100 μg to 1.6 μg per well, along with RBCs. PBS was used as a negative control for both of these assessments. Disruption of the lattice formation from the interaction of the in-pdm09 virus and RBCs by rHA1_53-269_ results in pellet formation, demonstrating that rHA1_53-269_ was able to competitively inhibit agglutination by the in-pdm09 viral antigen.

The binding activity of bacterially expressed rHA1_1-326_ and rHA1_53-269_ to sialic acid, the native influenza virus ligand, was evaluated in the hemagglutination assay using red blood cells (RBCs). Agglutination of RBCs is dependent on proper folding and/or exposure of the binding site, as this assay has been used as a surrogate for functional binding assays^13^. Since rHA1_53-269_ was unable to oligomerize, we anticipated that the hemagglutination assay with the rHA1_1-326_ and rHA1_53-269_ proteins would yield contrasting results. Consistent with a functional binding site and oligomerization, rHA1_1-326_ exhibited agglutination activity at concentrations greater than or equal to 12.5 µg or 250 μg/mL (Fig. 4A). In contrast, rHA1_53-269_ was unable to agglutinate RBCs.

To determine whether rHA1_53-269_ retained functional binding activity in the absence of oligomerization, a competition binding assay was designed using the standardized and inactivated H1N1 (in-pdm09). In this assay, rHA1_53-269_ binding activity was titered and incubated with 0.5% RBCs, followed by the addition of 4 hemagglutination units (HAUs) of the in-pdm09 control antigen (Fig. 4B). With progressively lower concentrations of rHA1_53-269_, RBC agglutination was proportionately restored. These results demonstrated that rHA1_53-269_ was able to compete with the H1N1 in-pdm09 viral antigen for the sialyloligosaccharides moieties in a specific, dose-dependent manner at concentrations greater than or equal to 12.5 µg or 250 μg/mL. Based on these collective findings, both rHA1_1-326_ and rHA1_53-269_ exhibited functional binding activity at the same concentration (≥250 μg/mL), regardless of oligomerization status.

### Oligomerization of HA1 contributes to the generation of neutralizing antibodies

The contribution of HA structure, including oligomerization, on the production of neutralizing antibodies was next investigated in mice immunized with one of the two rHA1 proteins (rHA1_1-326_ and rHA1_53-269_). The hemagglutination inhibition (HI) assay was used to detect and quantitate the levels of neutralizing antibodies that block the binding of HA1 to RBCs and subsequently prevent agglutination. Several highly regarded studies have demonstrated that hemagglutination inhibition titers were consistent with neutralization titers^17,18^, and other investigators have determined that a hemagglutination inhibition titer of 40 was equivalent to a neutralization titer of 20 in their assessments^19^. Further, the minimum neutralizing titer for protection from a vaccine was notably shown to occur at a HI titer that is ≥40 in humans^19^.

**Figure 5:**
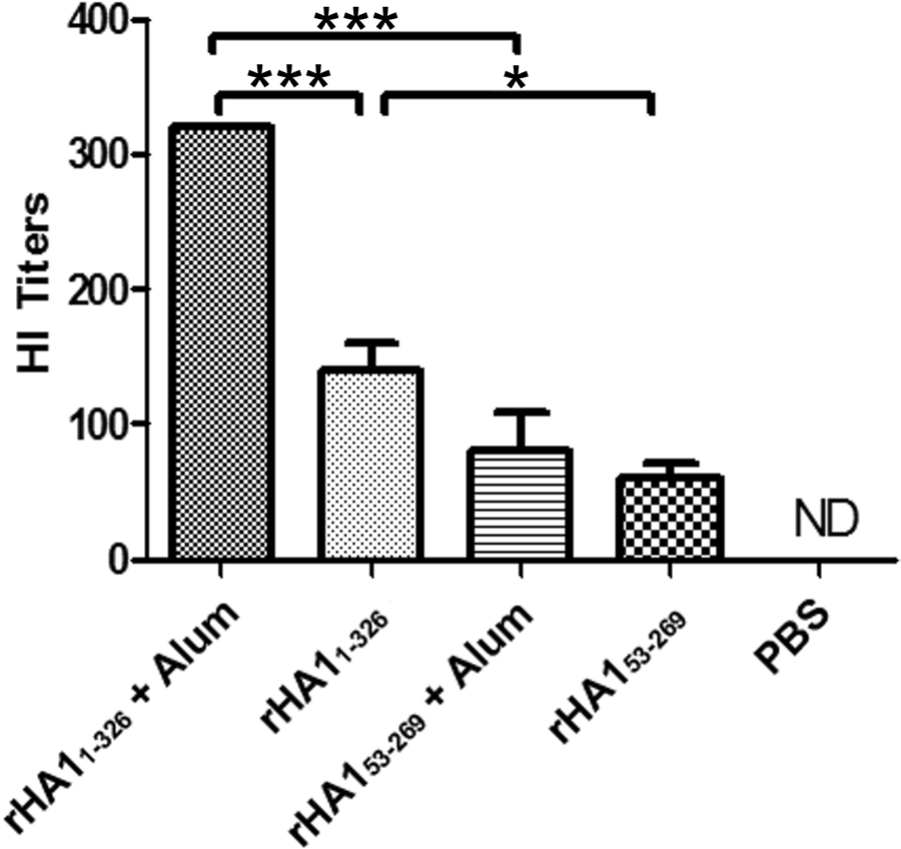
*In vivo* evaluation of the ability of rHA1_1-326_ and rHA1_53-269_ to produce neutralizing antibodies. The hemagglutination inhibition (HI) assay was used to detect and quantitate the levels of neutralizing antibodies that block the binding of the recombinant HA proteins to RBCs and subsequently prevent agglutination. The ability of the rHA1_1-326_ and rHA1_53-269_ proteins without or with the adjuvant Alum to neutralize antibodies is shown as the resulting HI titers, along with the significance. *p ≤ 0.01, ***p ≤ 0.001, ND = none detected.

In the current study, the presence of neutralizing antibodies in sera collected from the immunized mice was evaluated 21 days post-immunization using the HI assay. These evaluations showed that the most potent immune stimulation was achieved with rHA1_1-326_ and the adjuvant Alum (Fig. 5). The average neutralizing titer of rHA1_1-326_ with Alum was 320, while this recombinant HA1 protein without Alum elicited an average neutralizing titer of 140. rHA1_53-269_ with Alum resulted in an average neutralizing titer of 80, whereas without Alum, this truncated rHA1 elicited an average neutralizing titer of 60. Based on the production of neutralizing antibodies, rHA1_1-326_ stimulated statistically significant higher antibody levels than rHA1_53-269_ with adjuvant (*p* = 0.001) and without adjuvant (*p* = 0.01). Therefore, higher order HA1 oligomers substantially contributed to an increase in neutralizing antibodies.

### Oligomerization of recombinant HA contributes to protection against viral challenge

**Figure 6:**
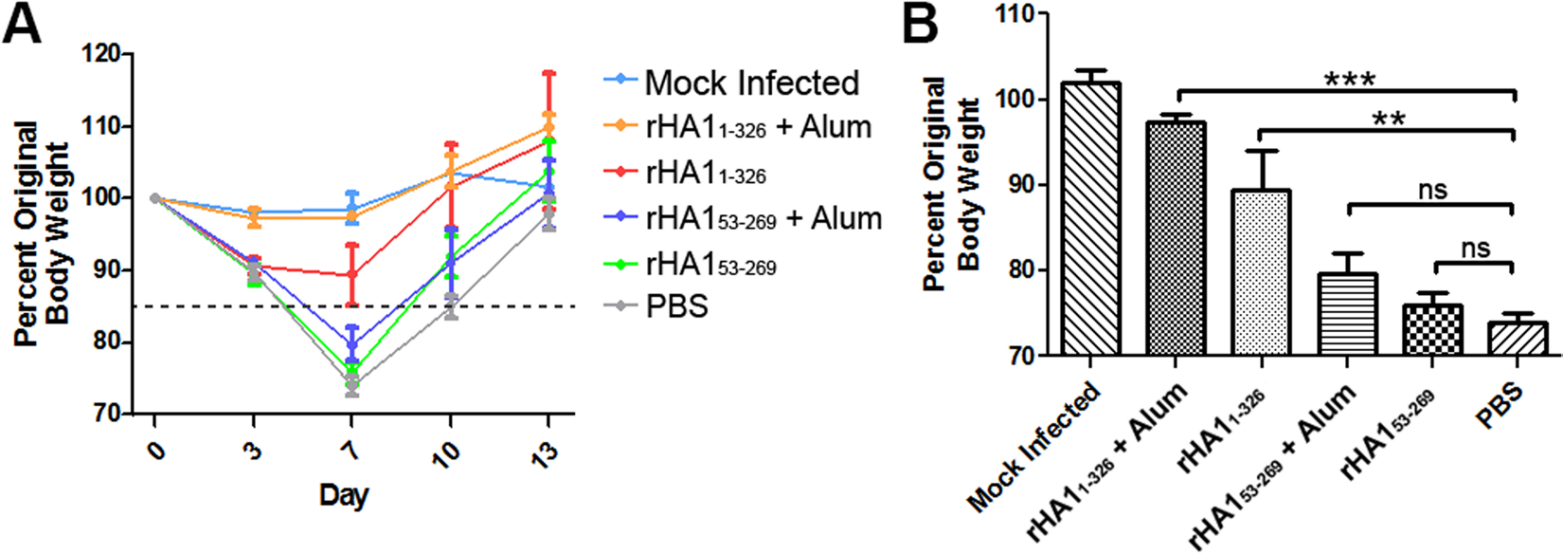
Ability of rHA1_1-326_ and rHA1_53-269_ to protect against an influenza infection as measured by body weight. (**A**) The daily change in body weight of mice from day 0 to the last day of the assessment at day 13 is presented as a percentage of the original baseline (day 0) weight, following exposure to H1N1 strain A/California/04/2009. Loss of more than 15% of original body weight (below dashed line) was designated as “unprotected” against viral challenge. (**B**) Comparison and significance of maximum body weight loss at day 7 post-exposure to H1N1 is shown. **p ≤ 0.005, ***p ≤ 0.001, ns = not significant.

Unvaccinated mice and mice vaccinated with one of the two rHA proteins (rHA1_1-326_ and rHA1_53-269_) were challenged with H1N1 strain A/California/04/2009, to determine whether oligomerization contributed to protection against viral challenge. Mice were infected with 100% minimum infectious dose (MID_100_) of the virus, and their weight was monitored for 13 days at the same time each day in order to measure infection severity. As shown in Fig. 6A, the changes in body weight were represented as a percentage of the original body weight at day 1 and the average weight of each group for each day was plotted. Any animal that lost more than 15% of original body weight was designated “unprotected” against viral challenge, which is denoted by a dashed line in Fig. 6A. Importantly, there was no obvious weight loss for the infected mice immunized with rHA1_1-326_ and Alum, nor the PBS mock-infected control group (Fig. 6A). For all the other experimental groups, the greatest weight loss was observed at day seven post-infection, with the PBS-immunized virus-infected control mice showing the highest weight loss of all the groups examined during the 13 day study. Recovery from viral challenge as measured by weight gain was seen at day 10 for the mice immunized with the rHA1_1-326_ without and with Alum, whereas the infected mice immunized with rHA1_53-269_ either without or with Alum and the PBS-immunized control mice did not recover until approximately day 13.

To assess the statistical significance for the protection observed, the average percentage of original body weight retained on day 7 post-infection was calculated for each group (Fig. 6B), since maximum weight loss occurred on this day (Fig. 6A). As shown in Fig. 6B, mice immunized with rHA1_1-326_ and adjuvant showed the highest level of protection when compared with PBS-immunized control mice (*p* < 0.001), with an average of 97.3% original body weight retained. Mice receiving rHA1_1-326_ without adjuvant retained an average 89.5% of their original body weight, which was significant when compared to PBS-immunized control mice (*p* < 0.005). rHA1_53-269_ immunized mice with and without adjuvant retained an average 79.7% and 76.0% of their original body weight on day 7 post-infection, respectively. However, body weights of rHA1_53-269_ immunized mice with or without adjuvant on day 7 post-infection were not statistically significant when compared to PBS-immunized control mice. Together these results demonstrated that oligomerized rHA1_1-326_ provided protection from viral challenge, whereas monomeric rHA1_53-269_ was unable to elicit the same response.

## Discussion

Although the demonstration of immunogenicity and the evaluation of the protective effect conferred by two recombinant HA subunit proteins, specifically rHA1_1-326_ and rHA1_53-269_, in the present study provided important information, the contribution of oligomerization on the ability of recombinant HA proteins to protect mice from viral challenge may have greater significance for new vaccine formulations. One factor that contributed to oligomerization was the screening and identification of an effective refolding buffer that aided the process and experimentally facilitated functional binding of both rHA1_1-326_ and rHA1_53-269_. The rHA1_1-326_ protein formed dimers and trimers, whereas rHA1_53-269_ did not form higher order oligomers, as confirmed by immunoblot analysis using non-reducing conditions and size exclusion chromatography. The amino acids 1–52 and 270–326 were determined to be critical for the ability of rHA1_1–326_ to oligomerize, indicating that the cysteines within these regions (amino acids 4, 42, 275, 279, and 303) play a crucial role in the oligomerization capability of rHA1_1-326_.

Our experimental model is consistent with the results obtained by others whereby recombinant H5 hemagglutinin (rH5) was expressed in a prokaryotic system and evaluated for the ability to protect from a H5N1 infection^13,15^. These studies determined that oligomerized rH5 elicited the production of higher neutralizing antibody titers than monomeric rH5, and importantly only oligomers of rH5 provided protection in viral challenge studies^13,15^. Our findings showed that although rHA1_1-326_ and rHA1_53-269_ differed in the ability to oligomerize, both recombinant HA1 proteins retained functional binding activity to sialic acid based on the hemagglutination and competitive binding assays (Fig. 4). These data indicated that a sufficient number of residues and/or epitopes were exposed for receptor binding. Moreover, both rHA1_1-326_ and rHA1_53-269_ elicited the production of neutralizing antibodies, as measured by HI assays; however, only rHA1_1-326_ without or with adjuvant protected against viral challenge, even though rHA1_53-269_ elicited the production of neutralizing antibodies at a HI ≥40.

In summary, our findings demonstrated that the presence of neutralizing antibodies, even at titers ≥40, does not guarantee protection *in vivo*, and that structural considerations (e.g., trimerization) should be incorporated into future recombinant vaccine designs. The low cost, ease of manipulation, and faster production times make prokaryotic expression systems an attractive alternative to cell culture or current egg-based vaccine platforms. As more influenza vaccine strategies are developed, identifying epitopes that confer protection against multiple influenza serotypes is critical to prevent future epidemic and pandemic influenza outbreaks.

## Materials and Methods

### Cloning of rHA1 fragments

Complementary DNA from the HA gene of a seasonal H1N1 influenza virus strain A/California/04/2009 was generated from a sequence-confirmed clinical isolate that was obtained from the Nebraska Public Health Laboratory. Two expression plasmids were constructed to encode either a long HA fragment (rHA1_1-326_) that includes residues 1-326, or a short HA fragment (rHA1_53-269_) containing residues 53-269. Both recombinant HA proteins were designed to contain all neutralizing domains, but only rHA1_1-326_ was designed to trimerize. rHA1_1-326_ and rHA1_53-269_ were amplified by PCR to contain an N-terminal His_6_ tag to facilitate purification. Amplicons were cloned in-frame into the pET28 expression vector (Novagen) and sequenced to confirm content. Plasmids were then transformed into *Escherichia coli* Rosetta-gami 2(DE3) pLysS (Novagen) for subsequent expression.

### Protein expression, purification, and refolding

Bacterial cultures were grown to an OD_600_ of 1.0 and then induced with 1 mM IPTG. Bacterial cells were lysed, filtered through a 0.22 μm filter, and purified with a His-Trap column on an FPLC (GE). Proteins were buffer-exchanged with a panel of “refolding buffers” (Thermo). The refolding buffer that contained 1.1 M guanidine, 440 mM L-arginine, 55 mM Tris, 21 mM NaCl, 0.88 mM KCl, 1 mM EDTA, 1 mM glutathione, and 1 mM glutathione disulfide (pH 8.2) was determined to induce protein refolding and trimerization. Following treatment with the refolding buffer, proteins were buffer exchanged into sterile PBS.

### Evaluating protein oligomer composition by immunoblot analysis and gel filtration

Immunoblot analyses of N-terminally histidine-tagged rHA1_1-326_ and rHA1_53-269_ were performed under both denaturing and non-denaturing conditions. Proteins were detected with an anti-His antibody (Sigma catalog no. H1029). Protein oligomerization was also evaluated by gel filtration using a HiPrep 16/60 Sephacryl S-300 HR column (GE), as recommended by the manufacturer.

### Hemagglutination assay

rHA1_1-326_ or rHA1_53-269_ (200 μg to 1.6 μg/well) was added to a V-bottom 96-well plate in triplicate and serially diluted 2-fold. Chicken RBCs (0.5%) were then added to each well and incubated for 30 minutes at room temperature, followed by agglutination assessment of the various recombinant HA dilutions.

### Competition binding assay

rHA1_53-269_ (100 μg to 1.6 μg/well) was added to a V-bottom 96-well plate in triplicate and titered. After adding 0.5% RBCs, samples were incubated for 5 minutes at room temperature. Next, 4 HAUs of H1N1 in-pdm09 control antigen (International Reagent Resource, catalog no. FR-187) was added to each well. The plate was agitated briefly and then incubated for 30 minutes at room temperature. The highest dilution resulting in inhibition was recorded as the hemagglutination inhibition titer or HI titer.

### Animal immunization and viral challenge

Protocols for the immunization and challenge experiments were submitted to and approved by the University of Nebraska Medical Center’s Institutional Animal Care and Use Committee. The University of Nebraska Medical Center is registered as a research facility with the United States Department of Agriculture under the Animal Welfare Act, and the care and use of animals for this research study was based on national guidelines and Federal Regulations. BALB/c 5-7 week old mice (Charles River, strain 028) were intramuscularly injected with PBS only or with PBS containing one of the two recombinant HA proteins (100 μg rHA1_1-326_ or rHA1_53-269_) either without or with Alum adjuvant (Sigma Imject), as per package insert instructions. Six animal groups were evaluated, including a negative control (PBS immunized, no viral challenge), and a positive control (PBS immunized, virus challenged). Experimental groups included rHA1_1-326_ without Alum, rHA1_1-326_ with Alum, rHA1_53-269_ without Alum, and rHA1_53-269_ with Alum. All groups included five mice (n = 5) to have sufficient statistical power. Sera were collected 21 days post-immunization in serum separator tubes (BD) and processed per manufacturer instructions. Nonspecific inhibitors and agglutinins in the sera were removed following the World Health Organization (WHO) protocols for the identification of influenza isolates^20^.

Twenty-eight days post-immunization, mice were anesthetized with 1.6 mg of ketamine and 0.4 mg of xylazine and then infected intranasally with 10^6^ TCID_50_ of H1N1 strain A/ California/04/2009 in a volume of 30 µL. All experimental groups were virus challenged with the MID_100_ of the virus, except for one group of mice (n = 5) that was “mock infected” with 30 µL of PBS (negative control). Animal weights were recorded daily for 13 days following viral challenge and compared to individual baseline (day 0) weights. Post-infection, animals were housed separately and weights were monitored at the same time daily for two weeks as a measure of infection severity. Although the presence of residual endotoxins as low as 0.1 ng/mL (1 endotoxin unit/mL or 1 EU/mL) are capable of causing symptoms, there was no significant difference in weight changes between the mice vaccinated with the mock PBS control and the purified rHA1_153-269_ protein, which did not confer significant protection either with or without adjuvant (Fig. 6B). No changes were made to the environment or the food and water available to each animal, and the animal studies were performed in two independent experiments.

### Hemagglutination inhibition assay

Treated sera (50 µL) were serially diluted 2-fold and then 25 µL (4 hemagglutination units or HAUs) of standardized and inactivated H1N1 pdm09 control antigen (International Reagent Resource, catalog no. FR-187) was added to all wells followed by brief agitation of the plate. After incubation of the plate at room temperature for 15 minutes, 50 µL of 0.5% RBCs was added to all wells. Plates were then read after 30 minutes of additional incubation at room temperature.

### Statistical analyses

Statistical analyses were completed using GraphPad Prism 5. One-way ANOVA with Tukey’s multiple comparison tests were used to determine significance for neutralizing antibody production and weight loss in the animal studies.

## Electronic supplementary material

Supplementary Figure S1
