# Supplementary material for: Oligomerization of bacterially expressed H1N1 recombinant hemagglutinin contributes to protection against viral challenge
Source: Sci Rep. 2018 Aug 7;8:11856. doi: 10.1038/s41598-018-30079-4 (PMC6081378; doi:10.1038/s41598-018-30079-4)

SUPPLEMENTARY INFORMATION

Oligomerization of bacterially expressed H1N1 recombinant hemagglutinin contributes to protection against viral challenge

Kuenstling, Tess E.[1], Sambol, Anthony R.[1,2], Hinrichs, Steven H.[1,2], and Larson, Marilynn A.[1*]

Supplementary Figure S1. Detection of H1N1 recombinant HA1 oligomers. Uncropped immunoblots of histidine-tagged recombinant HA1 proteins under denaturing and non-denaturing conditions and subsequent detection with an anti-His antibody. (**A**) Analysis of rHA1$_{1-326}$ (100 ng) under native conditions in elution buffer and without refolding buffer (U), and also after buffer exchange in refolding buffer (F). (**B**) Electrophoretic mobility of rHA1$_{1-326}$ (100 ng) with a predicted MW of 38.6 kDa under denaturing conditions (U). (**C**) Analysis of rHA1$_{53-269}$ (40 ng) with a predicted MW of 27.3 kDa under denaturing conditions (lane labeled "4_s"), and (**D**) under native conditions after buffer exchange in refolding buffer. L = Ladder or Molecular weight size markers in kDa and are shown to the left of the denaturing gel blots in panels B and C, F = Folded in refolding buffer, U = Untreated and eluted without refolding buffer.

A B C D

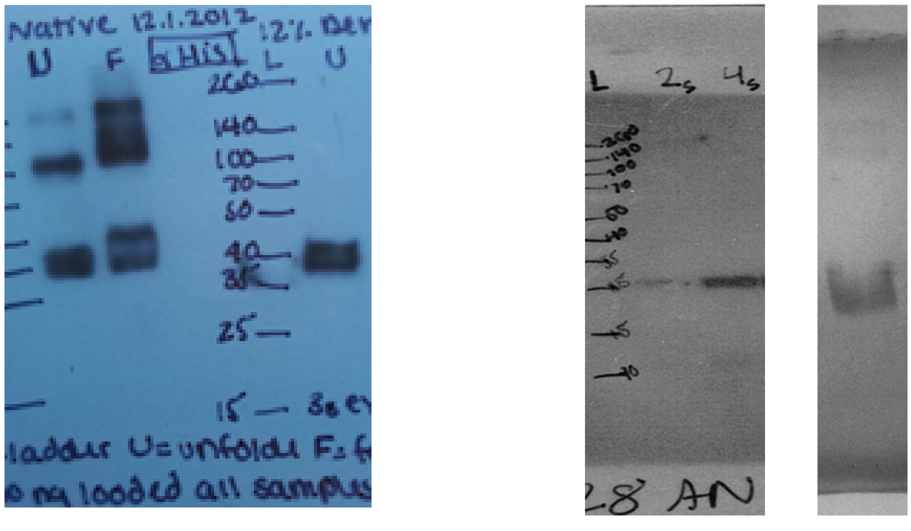

Supplement: Supplementary file 1 — Supplementary Figure S1 [file 41598_2018_30079_MOESM1_ESM.pdf]
